# Standardization of Laboratory Methods for the PERCH Study

**DOI:** 10.1093/cid/cix081

**Published:** 2017-05-27

**Authors:** Amanda J. Driscoll, Ruth A. Karron, Susan C. Morpeth, Niranjan Bhat, Orin S. Levine, Henry C. Baggett, W. Abdullah Brooks, Daniel R. Feikin, Laura L. Hammitt, Stephen R. C. Howie, Maria Deloria Knoll, Karen L. Kotloff, Shabir A. Madhi, J. Anthony G. Scott, Donald M. Thea, Peter V. Adrian, Dilruba Ahmed, Muntasir Alam, Trevor P. Anderson, Martin Antonio, Vicky L. Baillie, Michel Dione, Hubert P. Endtz, Caroline Gitahi, Angela Karani, Geoffrey Kwenda, Abdoul Aziz Maiga, Jessica McClellan, Joanne L. Mitchell, Palesa Morailane, Daisy Mugo, John Mwaba, James Mwansa, Salim Mwarumba, Sammy Nyongesa, Sandra Panchalingam, Mustafizur Rahman, Pongpun Sawatwong, Boubou Tamboura, Aliou Toure, Toni Whistler, Katherine L. O’Brien, David R. Murdoch

**Affiliations:** 1 International Vaccine Access Center, and; 2 Center for Immunization Research, Department of International Health, Johns Hopkins Bloomberg School of Public Health, Baltimore, Maryland;; 3 Kenya Medical Research Institute–Wellcome Trust Research Programme, Kilifi;; 4 Department of Infectious Disease Epidemiology, London School of Hygiene & Tropical Medicine, United Kingdom;; 5 Microbiology Laboratory, Middlemore Hospital, Counties Manukau District Health Board, Auckland, New Zealand;; 6 Center for Vaccine Innovation and Access, PATH, and; 7 Bill & Melinda Gates Foundation, Seattle, Washington;; 8 Global Disease Detection Center, Thailand Ministry of Public Health–US Centers for Disease Control and Prevention Collaboration, Nonthaburi;; 9 Division of Global Health Protection, Center for Global Health, Centers for Disease Control and Prevention, Atlanta, Georgia;; 10 InternationalCentre for Diarrhoeal Disease Research, Bangladesh (icddr,b), Dhaka and Matlab;; 11 Department of International Health, Johns Hopkins Bloomberg School of Public Health, Baltimore, Maryland;; 12 Division of Viral Diseases, National Center for Immunizations and Respiratory Diseases, Centers for Disease Control and Prevention, Atlanta, Georgia;; 13 Medical Research Council Unit, Basse, The Gambia;; 14 Department of Paediatrics, University of Auckland, and; 15 Centre for International Health, University of Otago, Dunedin, New Zealand;; 16 Division of Infectious Disease and Tropical Pediatrics, Department of Pediatrics, Center for Vaccine Development, Institute of Global Health, University of Maryland School of Medicine, Baltimore;; 17 Medical Research Council, Respiratory and Meningeal Pathogens Research Unit, and; 18 Department of Science and Technology/National Research Foundation, Vaccine Preventable Diseases Unit, University of the Witwatersrand, Johannesburg, South Africa;; 19 Center for Global Health and Development, Boston University School of Public Health, Massachusetts;; 20 International Centre for Diarrhoeal Disease Research, Bangladesh (icddr,b), Dhaka;; 21 Microbiology Department, Canterbury Health Laboratories, Christchurch, New Zealand;; 22 Department of Pathogen Biology, London School of Hygiene & Tropical Medicine, United Kingdom;; 23 Microbiology and Infection Unit, Warwick Medical School, University of Warwick, Coventry, United Kingdom;; 24 International Livestock Research Institute, Kampala, Uganda;; 25 Department of Clinical Microbiology and Infectious Diseases, Erasmus Medical Center, Rotterdam, The Netherlands;; 26 Fondation Mérieux, Lyon, France;; 27 Department of Biomedical Sciences, School of Medicine, University of Medicine, and; 28 Zambia Center for Applied Health Research and Development, Lusaka;; 29 Centre pour le Développement des Vaccins (CVD-Mali), Bamako;; 30 Cummings School of Medicine, University of Calgary,Canada;; 31 Department of Pathology and Microbiology, University Teaching Hospital, Lusaka, Zambia;; 32 Department of Medicine, Center for Vaccine Development, Institute of Global Health, University of Maryland School of Medicine, Baltimore; and; 33 Department of Pathology, University of Otago, Christchurch, New Zealand

**Keywords:** pneumonia, PERCH, laboratory, respiratory infection.

## Abstract

The Pneumonia Etiology Research for Child Health study was conducted across 7 diverse research sites and relied on standardized clinical and laboratory methods for the accurate and meaningful interpretation of pneumonia etiology data. Blood, respiratory specimens, and urine were collected from children aged 1–59 months hospitalized with severe or very severe pneumonia and community controls of the same age without severe pneumonia and were tested with an extensive array of laboratory diagnostic tests. A standardized testing algorithm and standard operating procedures were applied across all study sites. Site laboratories received uniform training, equipment, and reagents for core testing methods. Standardization was further assured by routine teleconferences, in-person meetings, site monitoring visits, and internal and external quality assurance testing. Targeted confirmatory testing and testing by specialized assays were done at a central reference laboratory.

The primary aim of the Pneumonia Etiology Research for Child Health (PERCH) study was to provide a contemporary picture of the microbial etiology of severe pneumonia in young children from developing countries [1]. One of the defining characteristics of the study was the use of a standard case definition and a rigorous training program to achieve standardization of case assessments and specimen collection [2]. The standardization of laboratory methods in the PERCH study was equally important to ensure comparability across study sites and for accurate and meaningful interpretation of pneumonia etiology results.

We have previously described the process leading to the PERCH diagnostic testing strategy [3–6]. Here we describe the laboratory methods used in PERCH and the procedures to ensure standardization and quality.

## PERCH LABORATORY STRUCTURE

Each PERCH study site included an established research laboratory with dedicated study staff overseen by 1 or more local laboratory managers. The PERCH laboratory director provided centralized oversight of laboratory activities across all sites. To build capacity at the sites, and in alignment with the priorities of the Bill & Melinda Gates Foundation, all PERCH testing was done locally, with the exception of quality assurance testing and a select subset of specialized assays, which were performed at the study reference laboratory (Canterbury Health Laboratories, Christchurch, New Zealand), which also served as the study specimen and isolate biorepository.

## PERCH RESEARCH SITES AND LABORATORIES

The PERCH site laboratories were located in Kilifi, Kenya; Basse and Banjul, The Gambia; Bamako, Mali; Lusaka, Zambia; Soweto, South Africa; Nakhon Phanom, Sa Kaeo, and Nonthaburi, Thailand; and Matlab and Dhaka, Bangladesh. Study sites were selected through an open, global site solicitation and selection process. As there was little support in the budget to expand existing infrastructure, preference was given to sites with well-established research laboratories and with experience in pediatric pneumonia studies.

## PERCH SPECIMEN COLLECTION AND TESTING ALGORITHM

Blood, nasopharyngeal and oropharyngeal (NP/OP) swabs, induced sputum (IS), and urine were collected from all PERCH cases at enrollment. Cases who were intubated had endotracheal aspirate collected in lieu of induced sputum. Gastric aspirate and pleural fluid specimens were collected when clinically indicated. At select sites, lung aspirates (Bangladesh, The Gambia, Mali, South Africa) and postmortem specimens (Thailand, South Africa) were also collected. Among PERCH controls, blood, NP/OP swabs, and urine were collected at enrollment at all sites.

Standardized recommendations for specimen storage prior to laboratory evaluation were provided in standard operating procedures (SOPs) (Table 1). All specimens were collected within walking distance or within a 2-hour drive of the study laboratory, with the exception of Matlab, Bangladesh, where specimens were transported once or twice a day from the field hospital to the laboratory in Dhaka, and Basse, The Gambia, where specimens were transported 1 to 2 times weekly to the Fajara laboratory for molecular testing. The transportation of all specimens was done under controlled temperature conditions, as stipulated in the study SOP.

**Table 1.** Specimen Transport and Storage Requirements

Specimen	Transport/Storage Conditions	Until
Blood culture	≤24 h, room temperature or according to manufacturer’s instructions	Placement in blood culture machine
Whole blood (EDTA and plain tubes)	<3 days, 2°C–8°C	Specimen separation
Urine	≤24 h, 2°C–8°C (≤2 h, room temperature)	Freezing (–70°C)
NP/OP swabs in viral transport medium	≤8 h, 2°C–8°C (≤2 h, room temperature)	Freezing (–70°C)
NP swab in STGG	<8 h, 2°C–8°C	Freezing (–70°C)
Induced sputum	≤24 h, 2°C–8°C (≤2 h, room temperature)	Inoculation onto culture media and other primary laboratory processing
Lung aspirate	≤24 h, 2°C–8°C (≤2 h, room temperature)	Inoculation onto culture media and other primary laboratory processing
Gastric aspirate	≤24 h, 2°C–8°C (≤15 min, room temperature)	Tuberculosis culture
Pleural fluid	≤24 h, 2°C–8°C (≤2 h, room temperature)	Inoculation onto culture media and other primary laboratory processing
Lung tissue	≤24 h, 2°C–8°C (≤2 h, room temperature)	Inoculation onto culture media and other primary laboratory processing

Abbreviations: EDTA, ethylenediaminetetraacetic acid; NP/OP, nasopharyngeal/oropharyngeal; STGG, skim milk, tryptone, glucose, and glycerin.

**Figure 1.**
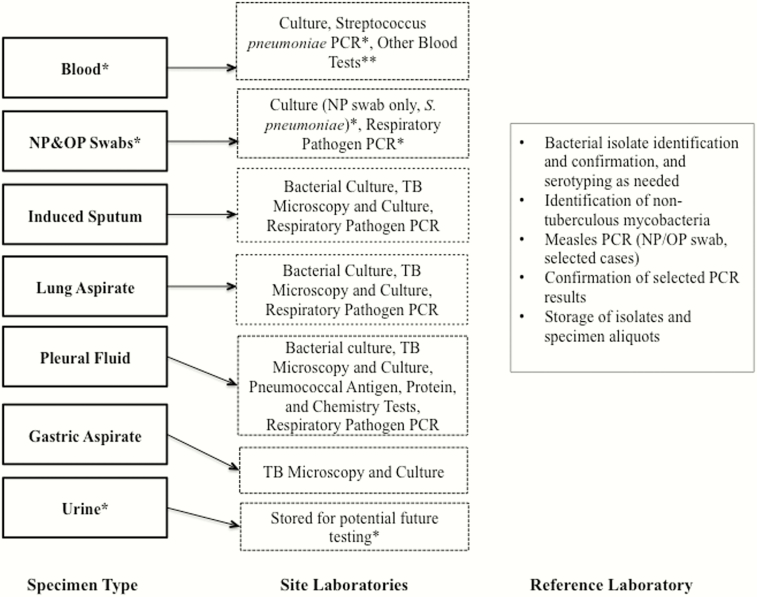
Pneumonia Etiology Research for Child Health (PERCH) study testing algorithm. *Applies to controls as well as cases. **May include complete blood count, C-reactive protein, malaria, human immunodeficiency virus, CD4, and/or thalassemia testing depending on site. Abbreviations: NP, nasopharyngeal; OP, oropharyngeal; PCR, polymerase chain reaction; TB, tuberculosis.

Standardized testing algorithms were developed for each body fluid type among cases and controls (Figure 1). Following testing, residual volumes of body fluid specimens were stored at –80°C to facilitate future research, with particular attention to bioethical considerations [7]. Bacterial isolates cultured in pure growth were stored at –80°C unless classified as contaminants.

Barcode labels were used on specimen collection containers, data collection forms, and laboratory requisition forms [8]. In the laboratory, specimen aliquots and isolates were archived in 2-mL sterile cryovials with silicone O-rings, labeled with thermostable labels, and inventoried using freezer management software [8]. Laboratory data were entered into an electronic data capture system to allow for real-time study monitoring [8].

## STANDARDIZED LABORATORY METHODS

### Molecular Diagnostics

To standardize molecular testing, an automated nucleic acid extraction platform and standardized polymerase chain reaction (PCR) assays, described below, were deployed across all study sites. The PERCH laboratory director reviewed all PCR results files prior to their inclusion in the study database.

### Respiratory Pathogen PCR

For PCR evaluation of respiratory specimens, we used the Fast-track Diagnostics Respiratory Pathogens 33 multiplex PCR kit (FTD Resp-33 kit) (Fast-track Diagnostics, Sliema, Malta). NP/OP specimens were collected in viral transport medium (universal transport medium [UTM], Copan Diagnostics, Bresica, Italy) and refrigerated at 2°C–8°C for a maximum of 8 hours, or frozen at –80°C prior to nucleic acid extraction. Induced sputum, pleural fluid, and lung aspirate specimens were collected in saline in universal containers and either refrigerated at 2°C–8°C for a maximum of 24 hours, or frozen at –80°C prior to nucleic acid extraction.

Total nucleic acid extraction was performed on respiratory specimens using the NucliSENS easyMAG platform (bioMérieux, Marcy l’Etoile, France). Four hundred microliters of each respiratory specimen (NP specimen in UTM, induced sputum aliquot in normal saline, pleural fluid aliquot, or lung aspirate aliquot) was eluted to a final volume of 60–110 μL nucleic acid. Prior to extraction, induced sputum specimens were digested with 1:1 dithiothreitol and incubated at ambient temperature until any mucus was broken down.

The FTD Resp-33 kit is a real-time PCR arranged in 8 multiplex groups for the detection of the following 33 viruses, bacteria, and fungi: influenza A, B, and C; parainfluenza viruses 1, 2, 3, and 4; coronaviruses NL63, 229E, OC43, and HKU1; human metapneumovirus A/B; human rhinovirus; respiratory syncytial virus A/B; adenovirus; enterovirus, parechovirus; bocavirus; cytomegalovirus; *Pneumocystis jirovecii*; *Mycoplasma pneumoniae*; *Chlamydophila pneumoniae*; *Streptococcus pneumoniae*; *Haemophilus influenzae* type b; *Staphylococcus aureus*; *Moraxella catarrhalis*; *Bordetella pertussis*; *Klebsiella pneumoniae*; *Legionella* species; *Salmonella* species; and *Haemophilus influenzae* species. The *K. pneumoniae* target was not used in any of the final analyses because of difficulties with assay specificity, as has been found elsewhere [9]. Positive, negative, and internal extraction controls were included in each run.

Quantitative PCR (qPCR) data were generated through the creation of standard curves using 10-fold serial dilutions of plasmid standards provided by FTD on an approximately quarterly basis at each study site, with calculation of pathogen density (copies/milliliter) from the sample cycle threshold (Ct) values. Because the results for the known standards were highly consistent across laboratories, standard curve data from all sites were pooled to create “standardized” standard curves for each pathogen target; data points beyond 2 standard deviations of the mean were excluded. Quantitative PCR was performed at each site using an Applied Biosystems 7500 (ABI-7500) platform (Applied Biosystems, Foster City, California). Cycling conditions were 50°C for 15 minutes, 95°C for 10 minutes, and 40 cycles of 95°C for 8 seconds followed by 60°C for 34 seconds.

### Pneumococcal PCR From Whole Blood Specimens

Whole blood samples were collected into a dedicated EDTA (ethylenediaminetetraacetic acid) tube and either refrigerated at 2°C–8°C for a maximum of 3 days, or frozen at –80°C prior to nucleic acid extraction. Total nucleic acid extraction was performed in batches with 200 μL of whole blood extracted and eluted to a final volume of 100 μL nucleic acid using the NucliSENS “specific B” protocol. Extracted DNA was frozen at –80°C until undergoing PCR for detection of the autolysin (*lytA*) gene.

*Streptococcus pneumoniae* nucleic acid was detected in whole blood using a qPCR assay based on a method from the US Centers for Disease Control and Prevention [10]. Mastermix containing 12.5 μL of Gene Expression Mastermix (Applied Biosystems, Life Technologies, California), 0.5 μL of each of the 10 μM forward and reverse primers and probe, 1 μL of molecular-grade water, and 10 μL of template DNA was used per reaction. Quantification standards consisting of *lytA* plasmids (Fast-track Diagnostics, Sliema, Malta) diluted 1:10 from 10^7^ copies/mL to 10^2^ copies/mL were run in triplicate on every plate. A no-template control, consisting of molecular-grade water, was likewise run in triplicate. Cycling conditions of 95°C for 10 minutes followed by 40 cycles of 95°C for 15 seconds and 60°C for 1 minute were applied on an ABI-7500 instrument. Exponential amplification curves with a Ct value of <40 cycles were considered positive and quantified using the standard curve.

### Confirmatory Testing of Selected PCR Targets at Reference Laboratory

All samples positive for *Bordetella pertussis* were tested for *Bordetella holmesii* [11]. A high proportion of samples positive for *H. influenzae* type b, especially from countries with Hib vaccine, called into question the specificity of that pathogen target. Consequently, we retested these positive samples with an established Hib assay [12] and used the results from this second assay in our analyses. Due to intermittent contamination of the nucleic acid extraction lysis buffer with *Legionella* species, samples positive for *Legionella* were retested at the reference laboratory [13].

Because few whole blood samples from Thailand and Bangladesh were positive for *lytA* PCR, we retested a random sample of 100 documented pneumococcal carriers (NP/OP specimens positive for *S. pneumoniae*) from both sites using the PERCH *lytA* PCR assay at the reference laboratory. No false negative blood results were found.

### Measles PCR

NP/OP swabs from all 33 cases with clinical signs or history of measles were tested for measles virus by PCR using the nucleoprotein gene target [14] at the reference laboratory. Suspected measles was defined as a history of measles in the past 3 months, measles rash at admission, or measles diagnosis at admission or discharge.

### Organism Identification Antimicrobial Susceptibility Testing

Organism identification was done according to standard microbiological methods that were documented in SOPs and clarified at each site at the outset; antimicrobial susceptibility testing followed the Clinical and Laboratory Standards Institute (CLSI) guidelines [15]. Antimicrobial susceptibility was tested using overnight growth of pure isolates, using the disk diffusion methodology when possible. *Streptococcus pneumoniae* isolates that had reduced susceptibility to penicillin by the oxacillin screen were tested by Etest (bioMérieux, low-dose strips) or a commercially available broth MIC method (TREK Diagnostic Systems) to measure minimum inhibitory concentrations (MIC) to penicillin. Enterobacteriaceae were screened for extended spectrum β-lactamase production using a cefotaxime (30 µg) disk and a ceftazidime (30 µg) disk. Zone sizes ≤27 mm for cefotaxime or ≤22 mm for ceftazidime were confirmed by the double disk diffusion test, following the CLSI guidelines. Organism identification was confirmed for each specimen type and is described below. Antibiotic susceptibility testing results were confirmed in a sample of 10% of isolates from all sites with 100% concordance of results.

### Blood Culture Processing

Blood cultures were incubated per manufacturer instructions using automated systems (BACTEC [Becton Dickinson, Sparks, Maryland] in Kenya, South Africa, The Gambia, Mali, and Zambia; BacT/ALERT [bioMérieux, Marcy l’Etoile, France] in Bangladesh and Thailand). Culture bottles were incubated within 24 hours of specimen collection. Alarm-positive culture specimens were plated on to 5% sheep or horse blood, chocolate, and MacConkey agar. Specimens were incubated for 5 days and then discarded. Specimens that were alarm-positive but subculture negative were tested using the BinaxNOW *Streptococcus pneumoniae* Antigen Card (Alere, Scarborough, Maine) if the Gram stain was either negative or revealed gram-positive cocci. Organisms were identified and antimicrobial susceptibility testing was performed according to CLSI methods. Any isolate classified as a contaminant was not stored; all other organisms were identified then stored at –80°C. Organisms were defined, a priori, as contaminants (Table 2). Of 195 stored blood culture isolates, 146 (72%) were shipped for confirmatory identification at the reference laboratory; of these, 121 (83%) were concordant with the original result. In the absence of strong evidence otherwise, organisms meeting the a priori contaminant definition were considered as contaminants. *Candida* species was also considered a blood culture contaminant if the patient recovered without antifungal treatment.

**Table 2.** Predefined Blood Culture Contaminants

Contaminant Organism^a^
*Aerococcus viridans*
*Streptococcus*, α-hemolytic (viridans)
*Bacillus* species
*Bacillus subtilis*
*Corynebacterium amycolatum*
*Corynebacterium kroppenstedtii*
*Corynebacterium macginleyi*
*Corynebacterium minutissimum*
*Corynebacterium* species
*Corynebacterium striatum*
*Corynebacterium ureolyticum*
*Corynebacterium pseudodiphtheriticum*
*Corynebacterium xerosis*
*Lactobacillus* species
*Leuconostoc* species
*Micrococcus* species
*Propionibacterium acnes*
*Propionibacterium avidum*
*Propionibacterium* species
*Staphylococcus capitis*
*Staphylococcus cohnii*
*Streptococcus constellatus*
*Staphylococcus epidermidis*
*Streptococcus gordonii*
*Staphylococcus hominis*
*Streptococcus intermedius*
*Staphylococcus kloosii*
*Streptococcus mitis*
*Staphylococcus muscae*
*Streptococcus mutans*
*Streptococcus oralis*
*Streptococcus parasanguis*
*Streptococcus salivarius*
*Streptococcus sanguinis (sanguis*)
*Staphylococcus sciuri*
*Staphylococcus simulans*
*Staphylococcus*, coagulase-negative
*Staphylococcus haemolyticus*
*Staphylococcus intermedius*
*Staphylococcus schleiferi*
*Staphylococcus warneri*
*Staphylococcus xylosus*

^a^All other organisms identified by blood culture were reviewed on a case-by-case basis to determine whether they were likely to be contaminants or the likely cause of the current hospitalization.

### NP Swab Processing for the Detection of Pneumococcal Carriage

NP swabs in skim milk tryptone-glucose-glycerin (STGG) medium were frozen at –80°C overnight, then thawed and processed using a broth-enrichment step to enhance pneumococcal carriage recovery [16, 17]. *Streptococcus pneumoniae* was identified by colony morphology, susceptibility to optochin, and bile solubility testing. Samples were inoculated onto 5% sheep or horse blood agar with 5 µg of gentamicin per milliliter and incubated at 35°C ± 2°C for 18–24 hours. Following subculture, each morphologically distinct pneumococcal colony was isolated and stored, with up to a maximum of 4 isolates per plate.

### Induced Sputum Culture

Efforts were made to process sputum specimens within 2, and no more than 24, hours following collection. Gram-stained smears were made from the most purulent portion of each induced sputum specimen. The number of epithelial cells and neutrophils per low-powered microscopic field were counted and recorded for the purpose of assessing specimen quality [18]. Microorganisms seen in the smear were described according to classic Gram stain morphotypes, with the number of bacterial morphotypes seen per high-powered field recorded to assist in interpretation of culture results.

The most purulent portion of each specimen was inoculated onto sheep or horse blood, chocolate, and MacConkey agars, streaked out using the 4-quadrant streaking method, and incubated at 35°C for 48 hours. Cultures were examined at 24 hours and 48 hours, and predominant organisms were identified and quantified according to the furthest quadrant with visible colonies (first quadrant = scanty; second quadrant = 1+; third quadrant = 2+; fourth quadrant = 3+). Background mixed oropharyngeal flora, including α-hemolytic streptococci, commensal *Neisseria*, coagulase-negative staphylococci, yeasts (except *Cryptococcus*), diphtheroids, and *Capnocytophaga* were quantified as a group but not identified further. Induced sputum specimens were also cultured for mycobacteria by standard liquid culture methods.

### Pleural Fluid and Lung Aspirate Culture

Gram stains were performed on all pleural fluid and lung aspirate specimens, and the number of leukocytes per low-powered field and bacterial morphotypes per high-powered field was recorded. Each specimen was cultured by plating onto chocolate and MacConkey agar and also inoculated in appropriate broth (blood culture bottles, tryptone soy broth, brain heart infusion, and brucella broth) and overnight incubation at 35°C–37°C. All plated and broth growth was examined at 24 hours and identified according to standard microbiological methods. Pleural fluid supernatant was assayed for protein and glucose and tested using the BinaxNOW *Streptococcus pneumoniae* Antigen Card. Pleural fluid and lung aspirate specimens were cultured for the presence of mycobacteria in liquid culture. Of 20 available pleural fluid and lung aspirate isolates, 12 (60%) were shipped to the reference laboratory for confirmation and all had their original organism identification confirmed.

### Pneumococcal Serotyping

Pneumococcal capsular serotyping was performed by the following methods: Quellung reaction (Zambia, South Africa), PCR deduction of pneumococcal serotypes [19] followed by Quellung reaction if there were mixed or ambiguous results by PCR (Thailand, Mali, The Gambia, Bangladesh), or latex agglutination at pool level with Quellung reaction for final typing and PCR confirmation of a subset of isolates as a quality control procedure (Kenya). Mixed or ambiguous results that could not be resolved at the study sites were serotyped by Quellung reaction at a reference laboratory (National Institute for Communicable Diseases, Johannesburg, South Africa or the Institute of Environmental Science and Research [ESR], Porirua, New Zealand). Serotyping for all pneumococcal isolates isolated from sterile sites as well as a sample of 50–75 pneumococcal isolates from the NP swab culture was verified by Quellung at the ESR laboratory.

### *Haemophilus* Serotyping

*Haemophilus influenzae* were identified at all sites using standard microbiological methods; serotype b was identified by slide agglutination. Additional (non type b) serotyping was performed in South Africa and The Gambia by slide agglutination. For all other sites, serotyping beyond type b was done by PCR at the reference laboratory (Canterbury Health Laboratories, Christchurch, New Zealand) [20].

### Antibiotic Bioassay

A bioassay was performed on enrollment serum samples, from all cases and controls, to detect antibiotic activity. A 6-mm filter paper disc was inoculated with 20 µL of serum and placed on a Mueller-Hinton plate seeded with a 0.5 McFarland suspension of a fully sensitive *Staphylococcus aureus* strain (ATCC 25923). Any zone of inhibited bacterial growth around the disc after 18–24 hours’ incubation was recorded as evidence of serum antibiotic activity.

### C-Reactive Protein

Serum from all cases was assayed for C-reactive protein (CRP). Samples from South Africa were assayed in country using CRP Gen3 Immunoturbidometric assay (Roche Diagnostics, Milan, Italy). All other samples were assayed at the reference laboratory in Christchurch, New Zealand, using CRP VARIO Immunoturbidometric assay (Roche Diagnostics). A subset of 682 control samples was assayed for CRP as part of an analysis to evaluate its diagnostic utility [21].

## LABORATORY INVESTIGATIONS DONE ACCORDING TO LOCAL PROTOCOL

### Other Blood Tests

A complete blood count was performed on all cases. Hemoglobin testing for controls was also carried out in The Gambia, Mali, and South Africa. Thalassemia testing for cases and controls was done in Thailand.

### Mycobacterial Culture

Culture of induced sputum and gastric aspirate specimens for mycobacteria was performed using liquid media in established tuberculosis testing laboratories at all sites. Antimicrobial susceptibility testing was performed on all *Mycobacterium tuberculosis* isolates. Isolates of nontuberculous mycobacteria were identified at the reference laboratory by 16S ribosomal RNA and rpoB sequencing [22–24].

### Malaria Testing

Malaria testing was performed by rapid antigen test or microscopy for all cases at sites with endemic malaria (Kenya, The Gambia, Mali, Zambia) and in South Africa when clinically indicated.

### Human Immunodeficiency Virus and CD4 Testing

Human immunodeficiency virus (HIV) testing was done on all cases at all sites apart from Bangladesh, and for controls at all Africa sites with the exception of The Gambia (which has an HIV infection prevalence of <2%). Testing was done by serum antibody assay, followed by PCR confirmatory antigen testing for cases and controls <18 months of age. In Zambia and South Africa, CD4 assessments were performed or collected from referral facilities for all HIV-infected cases and controls.

### Pneumocystis Testing

All respiratory specimens were tested for *P. jirovecii* nucleic acid by Fast-track PCR. Additionally, induced sputum, endotracheal aspirate, pleural fluid, and lung aspirate specimens were tested for *P. jirovecii* by immunofluorescence (South Africa) and toluidine blue staining (Zambia).

## INITIAL STANDARDIZATION AND TRAINING

Laboratory SOPs were developed in collaboration with site investigators for all core laboratory procedures. All sites underwent a period of training and pilot testing prior to study initiation. Following demonstration of successful performance, a site activation letter allowed formal study enrollment to commence [8]. The PERCH laboratory director, in conjunction with other team members, visited and evaluated each study laboratory prior to study piloting and provided advice on areas for additional improvement of facilities or training of staff.

Major equipment, including the nucleic extraction platform and PCR thermocyclers, was procured centrally and installed at each site laboratory. In addition, maintenance contracts were provided for the period of the study. Training on the nucleic acid extraction system was provided by bioMérieux at installation. Fast-track PCR training was provided in-person by Fast-track Diagnostics over a period of 3 days at each site. Trained staff received a certificate of completion and the site laboratory was required to pass an external quality assurance assessment before beginning molecular diagnostic testing on study samples.

Sites were trained on induced sputum slide reading at an initial training and again at a midstudy refresher training. A subset of approximately 10% of slides from each site were stored following reading and later sent to the reference laboratory where they were audited. Sites that were unable to send slides had a random sample checked by the laboratory director during study oversight visits.

Key SOPs were reviewed with laboratory scientists from all sites at an investigator meeting prior to study initiation, Training on the data capture system and freezer inventory software was provided remotely via a webinar prior to the start of the study.

### Ongoing Standardization and Quality Assurance

A working group including the laboratory director and representatives from all site laboratories was convened throughout the study to harmonize practices and troubleshoot problems at periodic investigator meetings and through regular teleconferences. A midstudy in-person refresher training on core laboratory procedures was conducted for all sites in August 2012. The laboratory director visited each study site at least twice over the course of the study to provide on-site monitoring. Laboratory quality indicators were monitored using the real-time data entry system and were used to identify areas for improvement over the duration the study [8]. Electronic laboratory data reports were generated from the database and reviewed at regular intervals by the laboratory director. In addition, digital PCR results files were rechecked at the reference laboratory to confirm accurate interpretation of PCR quantification curves. Discordant interpretations of results were discussed with the laboratories and corrected in the database.

### External Quality Control Assessments

An external quality assessment (EQA) program was set up by Fast-track Diagnostics to monitor performance of the Fast-track respiratory PCR and whole blood *lytA* PCR assays at each site. For the Fast-track respiratory EQA, laboratories were supplied with a series of 12 samples containing mixtures of plasmids at various concentrations at 3- to 4-month intervals. Each laboratory was required to test the samples using its routine FTD Resp-33 assay and standard procedures. Panels for the whole blood *lytA* PCR EQA were dispatched from FTD at the same frequency and included blinded plasmid samples containing the *lytA* target in a range of concentrations. For each round of EQA, an individual performance report was provided along with details of overall performance for all sites. Reports and practical feedback allowed participants to identify and resolve potential problems whilst monitoring the effectiveness of their laboratory quality assurance processes. Site-to-site variation was also assessed using these reports.

All sites were enrolled in an EQA program for the microbiological assessment of respiratory specimens, organized by the Royal College of Pathologists of Australasia Quality Assurance Programme. EQA panels were dispatched on a quarterly basis and consisted of simulated clinical specimens for the isolation of pathogens, bacterial identification and antimicrobial susceptibility testing. Most module shipments consisted of 2 specimens containing either a pure culture or a mixture simulating a clinical specimen with normal body flora. Following each dispatch, results were reviewed by the laboratory director and discussed with the site laboratories.

## DISCUSSION

We faced challenges in applying such a high level of standardization to a complex study across diverse research sites. Sites varied considerably in their prior level of experience with the PERCH laboratory methods and therefore required varying levels of assistance and oversight. Assuring that each laboratory had standard equipment in place and was comprehensively trained meant that the initiation of study enrollment and full specimen testing was delayed by weeks to months in some instances. Maintaining a high level of involvement and in-person oversight required regular travel by the laboratory director in addition to frequent communication by phone and email. Additionally, local approvals to ship specimens and isolates for confirmatory testing resulted in long delays in the reference laboratory receiving samples from some sites. Despite these challenges, we were able to achieve the highest methodological standards across a variety of circumstances, and demonstrated the ability to set up very complex molecular diagnostics in challenging environments. Achieving standards was a very positive motivator among laboratory staff, especially in laboratories that had not used international standards before. The value of regular feedback to the staff was evident in our study and we observed laboratory capacity and technical skills improve rapidly over a short period of time, with some of the laboratories without prior similar experience becoming the highest performers. Applying a high level of standardization required considerable effort in the study planning stage and throughout the enrollment and testing period, but in the end this effort was outweighed by our confidence that results were accurate and comparable across sites.

## CONCLUSIONS

PERCH was one of the largest pneumonia etiology studies ever undertaken, with a complex testing algorithm applied to >9500 individuals distributed over 9 enrollment centers in 7 different countries. Considerable efforts were made to perform as much of the laboratory testing at the study sites as possible, and to ensure cross-site standardization of testing methods. As well as providing confidence in the PERCH analyses, our experiences provide evidence that multisite studies involving extensive laboratory assessments and including complex molecular diagnostics can be undertaken at research sites in a variety of settings and circumstances, including those with limited prior experience.

## Supplementary Data

Supplementary materials are available at *Clinical Infectious Diseases online*. Consisting of data provided by the authors to benefit the reader, the posted materials are not copyedited and are the sole responsibility of the authors, so questions or comments should be addressed to the corresponding author.

## Supplementary Material

OTH_11_SupplementalMaterials

## Notes

***Author contributions.*** A. J. D. and D. R. M. drafted the manuscript. D. R. M. supervised all laboratory activities for the study. All authors were involved in the development of standard operating procedures, study conduct, data collection, and/or data management. All authors reviewed and approved the manuscript. A. J. D. and D. R. M. had final responsibility for the decision to submit for publication.

***Acknowledgments.*** We offer sincere thanks to the patients and families who participated in this study. We acknowledge the significant contributions of the PERCH Study Group and all PERCH investigators. We offer our gratitude to the members of the Pneumonia Methods Working Group and PERCH Expert Group for their time and lending expertise to assist the PERCH Study Group. See Supplementary Materials for a full list of names.

***Financial support.*** PERCH was supported by the Bill & Melinda Gates Foundation (grant number 48968 to the International Vaccine Access Center, Department of International Health, Johns Hopkins Bloomberg School of Public Health). J. A. G. S. was supported by a clinical fellowship from the Wellcome Trust of Great Britain (award number 098532).

***Supplement sponsorship.*** This article appears as part of the supplement “Pneumonia Etiology Research for Child Health (PERCH): Foundational Basis for the Primary Etiology Results,” sponsored by a grant from the Bill & Melinda Gates Foundation to the PERCH study of Johns Hopkins Bloomberg School of Public Health, Baltimore, Maryland.

***Potential conflicts of interest.*** K. L. O. has received grant funding from GSK and Pfizer and participates on technical advisory boards for Merck, Sanofi Pasteur, PATH, Affinivax, and ClearPath. M. D. K. has received funding for consultancies with Merck, Pfizer, and Novartis, and grant funding from Merck. L. L. H. has received grant funding from Pfizer and GlaxoSmithKline. K. L. K. has received grant funding from Merck Sharp & Dohme. S. A. M. has received honoraria for advisory board membership from the Bill & Melinda Gates Foundation, Pfizer, Medimmune, and Novartis; has received institutional grants from GSK, Novartis, Pfizer, Minervax, and the Bill & Melinda Gates Foundation; and has served on speaker’s bureaus for Sanofi Pasteur and GSK. All other authors report no potential conflicts. All authors have submitted the ICMJE Form for Disclosure of Potential Conflicts of Interest. Conflicts that the editors consider relevant to the content of the manuscript have been disclosed.
