# Supplementary material for: Standardization of Laboratory Methods for the PERCH Study
Source: Clin Infect Dis. 2017 May 27;64(Suppl 3):S245–52. doi: 10.1093/cid/cix081 (PMC5447855; doi:10.1093/cid/cix081)
Supplement: OTH_11_SupplementalMaterials [file cix081_suppl_OTH_11_SupplementalMaterials.pdf]

Acknowledgements:

***PERCH Study Group*. Johns Hopkins Bloomberg School of Public Health, Baltimore, Maryland**: Katherine L. O'Brien (PI), Orin S. Levine (Former PI, current affiliation Bill & Melinda Gates Foundation, Seattle, Washington**),** Maria Deloria Knoll (co-PI), Daniel R. Feikin (joint affiliation with Centers for Disease Control and Prevention, Atlanta, Georgia), Andrea N. DeLuca, Amanda J. Driscoll, Wei Fu, Laura L. Hammitt, Melissa M. Higdon, E. Wangeci Kagucia, Ruth A. Karron, Mengying Li, Daniel E. Park, Christine Prosperi; **The Emmes Corporation, Rockville, Maryland:** Nora L. Watson, **University of Otago, Christchurch, New Zealand**: David R. Murdoch; **ICDDR, b, Dhaka and Matlab, Bangladesh**: W. Abdullah Brooks (site PI), Hubert P. Endtz, Khalequ Zaman, Doli Goswami, Lokman Hossain, Yasmin Jahan, Hasan Ashraf; **Medical Research Council, Basse, The Gambia**: Stephen R. C. Howie (site PI), Bernard E. Ebruke, Martin Antonio, Jessica McLellan, Eunice Machuka, Arifin Shamsul, Syed M.A. Zaman, Grant Mackenzie; **KEMRI-Wellcome Trust Research Programme, Kilifi, Kenya**: J. Anthony G. Scott (site PI and PERCH co-PI), Juliet O. Awori, Susan C. Morpeth, Alice Kamau, Sidi Kazungu; **Division of Infectious Disease and Tropical Pediatrics, Department of Pediatrics, Center for Vaccine Development, Institute of Global Health, University of Maryland School of Medicine, Baltimore, Maryland and Centre pour le Développement des Vaccins (CVD-Mali), Bamako, Mali**: Karen L. Kotloff (site PI), Milagritos D. Tapia, Samba O. Sow, Mamadou Sylla, Boubou Tamboura, Uma Onwuchekwa, Nana Kourouma, Aliou Toure; **Respiratory and Meningeal Pathogens Research Unit, University of the Witwatersrand , Johannesburg, South Africa**: Shabir A. Madhi (site PI), David P. Moore, Peter V. Adrian, Vicky L. Baillie, Locadiah Kuwanda, Azwifarwi Mudau, Michelle J. Groome; **Thailand Ministry of Public Health – U.S. CDC Collaboration, Nonthaburi, Thailand**: Henry C. Baggett (site PI), Somsak Thamthitiwat, Susan A. Maloney (former site PI), Charatdao Bunthi, Julia Rhodes, Pongpun Sawatwong, Pasakorn Akarasewi (site co-PI, Ministry of Public Health); **Boston University School of Public Health, Boston, Massachusetts and University Teaching Hospital, Lusaka, Zambia**: Donald M. Thea (site PI), Lawrence Mwananyanda, James Chipeta, Phil Seidenberg, James Mwansa, Somwe wa Somwe, Geoffrey Kwenda; **Canterbury Health Laboratory, Christchurch, New Zealand:** Trevor P. Anderson, Joanne Mitchell.

PERCH Expert Group. William C. Blackwelder, Harry Campbell, John A. Crump, Adegoke Falade, Menno D. de Jong, Claudio Lanata, Kim Mulholland, Shamim Qazi, Cynthia G. Whitney.

Pneumonia Methods Working Group. Robert E Black, Zulfiqar A Bhutta, Harry Campbell, Thomas Cherian, Derrick W Crook, Menno D de Jong, Scott F Dowell, Stephen M Graham, Keith P Klugman, Claudio F Lanata, Shabir A Madhi, Paul Martin, James P Nataro, Franco M Piazza, Shamim A Qazi, and Heather J Zar.

Supplemental Materials: PERCH Laboratory Methods

PERCH Contributors:

Bangladesh: Kamrun Nahar, Arif Uddin Sikdir, Sharifa Yeasmin, Dilruba Ahmed, Muhammad Ziaur Rahman, Muhammad Yunus, Muhammad Al Fazl Khan, Muhammad Jubayer Chisti, Abu Sadat Muhammad Sayeem, Shahriar Bin Elahi, Mustafizur Rahman; **The Gambia:** Michel Dione, Emmanuel Olutunde, Peter Githua, Ogochukwu Ofordile, Rasheed Salaudeen, David Parker; **Kenya:** Shebe Mohamed, Siti Ndaa, Micah Silaba, Neema Muturi, Angela Karani, Sammy Nyongesa, Anne Bett, Daisy Mugo, Salim Mwarumba, Robert Musyimi, Andrew Brent, James Nokes, David Mulewa, Joyce Sande, John Odhiambo, Joshua Wambua, Nuru Kibirige, Caroline Mulunda, Hellen Mjalla, Norbert Katira, Karen Dama, Loice Masha, Christine Mutunga, Mwanajuma Ngama, Stephen Mangi, Riziki Anthony, Mwarua Yubu, Elijah Wakili, Benson Katana, Shoboi Mgunya, Emmanuel Mumba, Benedict Mver, George Kuria, Felix Githinji, Norbert Kihuha, Boniface Jibendi, Tahreni Bwanaali, Agustus Kea; **Mali:** Nana Kourouma, Aliou Toure, Mahamadou Diallo, Breana Barger-Kamate, Mariam Samake, Seydou Sissoko, Abdoul Aziz Maiga, Mariam Samake, Toumani Sidibe, Mariam Sylla, Aziz Diakite, Bassirou Diarra; **South Africa:** Azwidihwi Takalani, Andrea Hugo, Susan Nzenze, Ndulela Titi, Mmabatho Selela, Malebo Motiane, Minah Nkuna, Nonhlanhla Tsholetsane, Sibonsile Moya, Debra Katisi, Tondani Netshishivhe, Lerato Mapetla, Gudani Singo, Simphiwe Gasa, Cece Mgenge, Nozipho Mthunzi, Nombulelo Monedi, Tanja Adams, Shafeeka Mangera, Jeannette Wadula, Peter Tsaagane, Jenifer L. Vaughan, Sakina Loonat, Martin Hale, Sugeshnee Pather, Mariëtte Middel, Siobhan Trenor, Palesa Morailane, Ntombi Maya, Rene Sterley, Charné Combrinck, Given Malete, Lerato Qoza, Grizelda Liebenberg, Hendrik van Jaarsveld, Zunaid Kraft, Lisa-Marie Mollentze, Lourens Combrinck, Tsholofelo Mosome; **Thailand:** Sununta Henchaichon, Dr. Tussanee Amornintapichet, Dr. Somchai Chuananont, Toni Whistler, Juraiporn Ratanodom, Patranuch Sapchookul, Ornuma Sangwichian, Sirirat Makprasert, Manoon Hirunsalee, Possawat Jorakate, Anek Kaewpan, Duangkamol Siludjai, Apiwat Lapamnouysup, Dr. Wantana Paveenkittiporn, Waraporn Ubonphen, Dr. Peera Areerat, Ms.Yupapan Wannachaiwong, Ms Tewa Faipet, Ms Punnat Natnarakorn, Ms Ahchanan Sacharone, Mr.Winai Makmool, Ms. Kanlaya Sornwong, Ms. Promporn Sansuriwong, Ms. Ratchanida Potiya, Ms. Wasana Hongsawong, Ms.Wipa Matchaikhen, Ms. Thatsanawan Chaiyabil, Ms.Piyapai Wannarach, Ms Chamaiporn Wadeesirisak, Mr. Yuttapong Norapet, Mattana Bangkung, Mr. Barameht Piralam, Sathapana Naorat, Anchalee Jatapai, Prasong Srisaengchai, Dr. Leonard Peruski, Ms.Dawan Phaensoongnoen, Ms.Tussaaorn Klangprapan, Ms.Narawadee Dumrongdee, Ms.Atchara Srithongkham, Mr. Piyawut Noinont, Ms. Pornthip Kamlee, Ms.Siyapa Mongkornsuk; **Zambia:** Justin Mulindwa, Musaku Mwenechanya, John Mwaba, Magdalene Mwale, Julie Duncan, Kazungu Siazele, Muntanga Mapeni, Emily Hammond; **Canterbury Health Laboratory, Christchurch, New Zealand:** Rose Watt, Shalika Jayawardena; **The Emmes Corporation, Rockville, Maryland:** Mark Wolff, Megan Sanza, Omid Neyzari.
